# The respective contributions of visual and proprioceptive afferents to the mirror illusion in virtual reality

**DOI:** 10.1371/journal.pone.0203086

**Published:** 2018-08-30

**Authors:** Marion Giroux, Julien Barra, Issam-Eddine Zrelli, Pierre-Alain Barraud, Corinne Cian, Michel Guerraz

**Affiliations:** 1 Univ. Grenoble Alpes, Univ. Savoie Mont Blanc, CNRS, LPNC, Grenoble, France; 2 Univ. Grenoble Alpes, Univ. Savoie Mont Blanc, CNRS, CHU Grenoble Alpes, Grenoble INP, TIMC-IMAG, Grenoble, France; 3 Institut de Recherche Biomédicale des Armées, Brétigny sur Orge, France; University of Muenster, GERMANY

## Abstract

The reflection of passive arm displacement in a mirror is a powerful means of inducing a kinaesthetic illusion in the static arm hidden behind the mirror. Our recent research findings suggest that this illusion is not solely visual in origin but results from the combination of visual and proprioceptive signals from the two arms. To determine the respective contributions of visual and proprioceptive signals to this illusion, we reproduced the mirror paradigm in virtual reality. As in the physical version of the mirror paradigm, one of the participant’s arms (the left arm, in our study) could be flexed or extended passively. This movement was combined with displacements of the avatar’s left and right forearms, as viewed in a first-person perspective through a virtual reality headset. In order to distinguish between visual and proprioceptive contributions, two unimodal conditions were applied separately: displacement of the avatar’s forearms in the absence of physical displacement of the left arm (the visual condition), and displacement of the left forearm while the avatar’s forearms were masked (the proprioceptive condition). Of the 34 female participants included in the study, 28 experienced a kinaesthetic mirror illusion in their static (right) arm. The strength of the illusion (expressed in terms of speed and duration) evoked by the bimodal condition was much higher than that observed in either of the two unimodal conditions. Our present results confirm that the involvement of visual signals in the mirror illusion—often considered as a prototypic visual illusion—has been overstated. The mirror illusion also involves non-visual signals (bilateral proprioceptive-somaesthetic signals, in fact) that interact with the visual signals and strengthen the kinaesthetic effect.

## Introduction

The nature of most events humans are confronted with, rarely, if ever, consists of a unimodal sensory signal. In addition, no sensory system is precise and accurate enough to provide a robust percept under all conditions. To overcome this uni-sensory limitation, the central nervous system can combine and integrate all available signals for a more robust percept [1]. Despite these evidences, vision remains often considered in sensory neurosciences to dominate perception, particularly when the spatial domain is concerned [2–4]. The fact that illusory movements of the whole body or of individual body parts can be evoked by visual manipulation attests, indeed, of the importance of visual signals, particularly when kinaesthesia (the sense of movement) is concerned. For instance, participants confronted with the motion of a large part of the visual scene (such as when a nearby train starts to move) often report a sensation of body displacement in the opposite direction [5–7]. The illusory movement can be limited to a body segment (the participant’s hand, for example) when the rotating visual scene is displayed immediately underneath or around the said segment [8]. The mirror illusion evoked by the reflection of someone’s own moving hand through a mirror positioned in the sagittal plan is particularly fascinating. The “virtual hand” stands in for the unseen one, and displacement gives the appearance of symmetrical, bimanual movements; the unseen static hand (hidden behind the mirror) is perceived to move [9,10]. In addition to its scientific interest, the mirror illusion has been extensively used in rehabilitation wards to promote recovery from hemiplegia or to alleviate phantom limb pain in amputees [11].

Visual signals undoubtedly constitute an essential component of the mirror illusion, as demonstrated by Ramachandran and Altschuler’s 2009 study [9]. These researchers suggested that mirror neurons (which are known to fire when someone performs a motor task or simply observes someone else performing the task [12]) have a key role in the mirror illusion and mirror therapy. These neurons provide a neurophysiological mechanism through which visual signals are interpreted as proprioceptive or tactile signals and then elicit a mirror illusion. However, the enthusiasm for the “mirror” illusion should not make us forget that, as for most events humans are confronted with, non-visual signals accompany the reflected hand in the mirror, and might also contribute to the illusion. In a recent series of experiments, we confirmed that the mirror’s reflection of passive arm displacement is a powerful means of inducing a kinaesthetic illusion [10,13]. However, we also showed that masking the muscle proprioceptive afferents (by co-vibrating the biceps and triceps antagonist muscles at 40Hz) of the passively displaced reflected arm reduces the intensity of the kinaesthetic illusion perceived in the unseen arm behind the mirror [14]. We also showed that the so called mirror illusion can even survive visual occlusion [15]. In that study, once participants experienced the illusion that their hidden arm was moving, then either their view of the mirror was occluded (using occlusive glasses) and /or arm displacement was prevented. We found that once the kinaesthetic illusion was evoked, it could survive visual occlusion for a few seconds and decayed slowly as long as the contralateral arm was still moving [15]. Taken as a whole, these results (i) suggest that the mirror illusion is not purely visual but results from multisensory integration, and (ii) confirm that proprioceptive afferents are bimanually integrated (see also [16] and [17]).

Since the virtual moving mirror hand in the mirror paradigm is systematically accompanied by non-visual signals related to the real, moving hand (the image of which is reflected in the mirror), it is particularly difficult to determine the involvement of visual signals *per se*. To overcome this methodological issue, we designed an experiment in which the mirror paradigm was reproduced in virtual reality. The rationale was to separate one modality from another (vision of the avatar from proprioception of the passively moved arm) in order to better understand the respective contribution of these two modalities in the so called mirror illusion. Only passive arm displacements were considered in the present experiment. Participants were equipped with position sensors located on their forearms, which enabled us to couple real displacements of participant’s forearms with those of the avatar’s forearms viewed via a virtual reality headset. As in the physical version of the mirror paradigm, one of the participant’s forearms (the left one, in the present study) could be passively flexed or extended. These passive movements were combined with displacements of the avatar’s left and right forearms. Vision and proprioception were therefore combined in this sensory condition (bimodal condition). In order to distinguish between visual and proprioceptive contributions to the kinaesthetic mirror illusion, two unimodal conditions were applied separately: displacement of the avatar’s forearms in the absence of physical displacement of the left forearm (the visual unimodal condition), and displacement of the left forearm while the avatar’s forearms were masked (the proprioceptive unimodal condition). Participants were required in each of these three sensory conditions (bimodal, vision and proprioception) to verbally rate the direction, the speed and the duration of the illusory displacement experienced in the right arm. Our results showed that the kinaesthetic mirror illusion arises from the integration of visual and bilateral proprioceptive afferents.

## Method

### Participants

Since the avatar used here was a female, only females were included in the experiment. Thirty-four healthy adult females took part in the experiment (mean ± standard deviation (SD) age: 20.5 ± 4.2 years). None of the study participants had a history of visual, proprioceptive or neuromuscular disorders. All the participants provided their written, informed consent prior to initiation of the experiment. The study was performed in accordance with the ethical standards laid down in the 1964 Declaration of Helsinki and was approved by the local independent ethics committee (C.E.R.E.U.S _2018_4, Savoie Mont Blanc University, Chambéry, France).

### Material

A head-mounted display (HMD) designed for immersive virtual reality environments (Oculus Rift; Oculus VR, Irvine, CA, US) was used in the present experiment. The HMD included built-in, real-time tracking mechanisms to estimate the head’s position and orientation; this feature enables perspective correction and allows the user to move his head freely in a room-sized environment. The HMD also features supra-aural 3D spatial audio headphones, through which white noise was played during the experiment. The virtual reality scenario was developed with Unity software (Unity Technologies SF, US) and ran on a computer equipped with a MSI Geforce GTX 980 Gaming 4G graphics card (Micro-Star International, Taïwan) and an Intel Core i7–4790K processor (Intel Corporation, US). The virtual environment consisted in a 3D room with checkerboard pattern walls covered and a clock positioned on the wall facing the participant. The avatar was a seated female with the elbows positioned on a virtual table, viewed from a first-person perspective. Displacements of the avatar’s forearms were driven in real time by the displacements of the participant’s forearms, as sensed by an electromagnetic motion capture system (Liberty^™^, Polhemus, Colchester, VT, US) at a frequency of 240 Hz.

**Fig 1:**
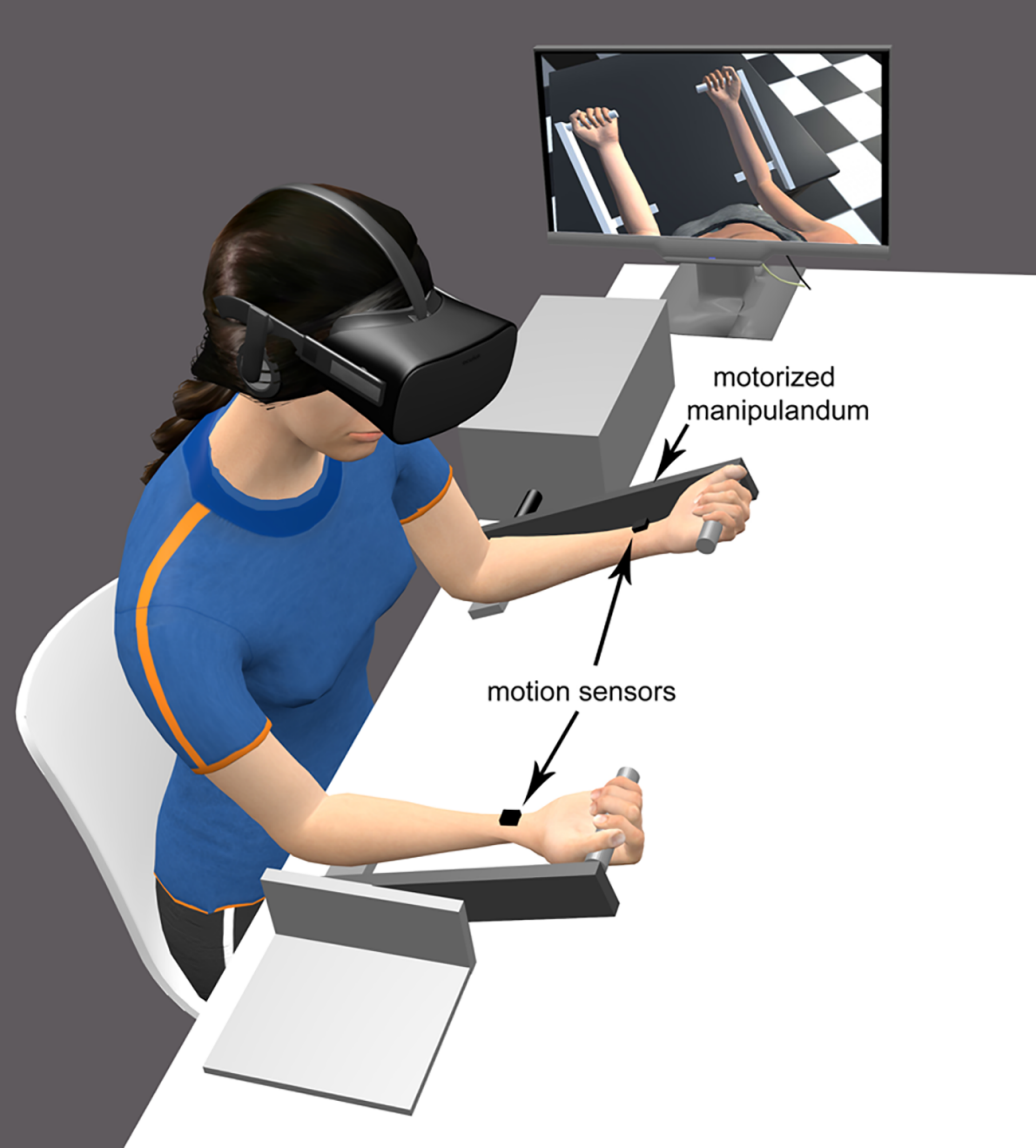
The experimental setup, in which the seated participant wore a virtual reality HMD. The experimenter’s monitor replicated the participant’s view of the virtual scene.

The participant sat at a table with his forearms placed forwards, in parallel and in the sagittal plan. During the experiment, each of the forearms was held by a manipulandum aligned with the participant’s shoulder. In order to avoid wrist movements, participants wore splints on each hand on which the sensors were positioned. Each manipulandum consisted of a wooden arm (on which the participant positioned his forearm) and a handgrip at the end of the wooden arm. The right manipulandum was fixed, whereas the left manipulandum was fitted with a low-noise synchronous DC motor (24V, Maxon with planetary Maxon reductor 1296:1 Switzerland) and could flex or extend (via a remote control) the participant’s left forearm from the initial starting position (Fig 1). The manipulandum’s angular speed was set to 3.8°/s. The participant’s left forearm was positioned on the manipulandum so that the latter’s rotational axis coincided with that of the elbow joint. The two manipulanda were replicated in the virtual environment, with the avatar’s hands holding the handgrips.

### Procedure

Wearing the virtual reality HMD, the participant sat at a table, with his elbows on the table. Before each block of trials, the participant had to actively flex and extend his forearms for a minute by alternating phase and antiphase movements. During this appropriation phase, the avatar’s forearms faithfully reproduced the participant’s real movements, as captured by the electromagnetic motion capture system. After the first period of appropriation, the participant filled out a brief, French-language questionnaire containing 4 of the 24 item (1, 5, 14, 15) from Witmer et al.’s presence questionnaire [18]. The latter contains a set of assertions, and each item is scored on a seven-point Likert scale. A mean score was computed for each participant. The score for the question “14” from Witmer et al.’s presence questionnaire was inverted for computational purposes. Witmer et al.’s presence questionnaire tests the extent of virtual immersion and checks that the participant has a feeling of “presence” in the virtual environment. Each of the 34 participants had a score attesting to a feeling of presence. The mean ± SD score (on the 7-point scale) for the four assertions was 6.3 ± 0.6, with a lowest score of 4.5. This appropriation phase was repeated after each block of four experimental trials.

**Fig 2:**
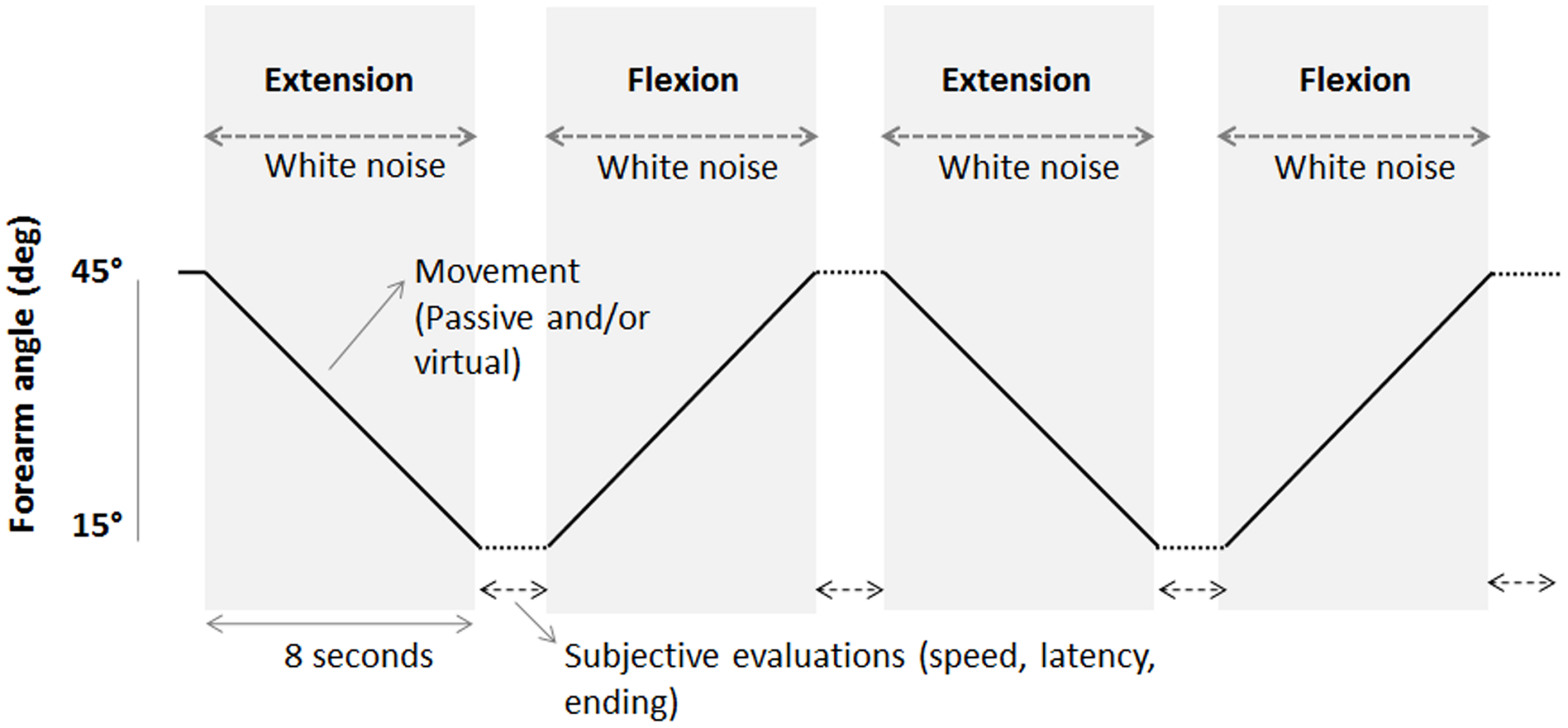
A block of four trials. The solid line represents the displacement of the left forearm through flexion-extension of the left arm by the motorised manipulandum.

Once the first appropriation phase had been completed, the participant’s forearms were positioned on the manipulanda. The right arm remained static and was held at 30° to the horizontal plane. The left arm was positioned at either 15° or 45° relative to the horizontal plane before each sequence of four trials. Following a baseline period of a few seconds, the left arm could be passively moved with an angular amplitude of 30°, a constant angular speed of 3.8°/s and thus a duration of 8 s. The sequence of movements consisted of four movements with two flexion and extension movements (Fig 2). Each sequence of movements therefore included four trials. During the interval between each movement, the participant subjectively estimated the direction, speed and duration of any illusory movement (see below for details). Participants were told not to resist the passive movements.

**Fig 3:**
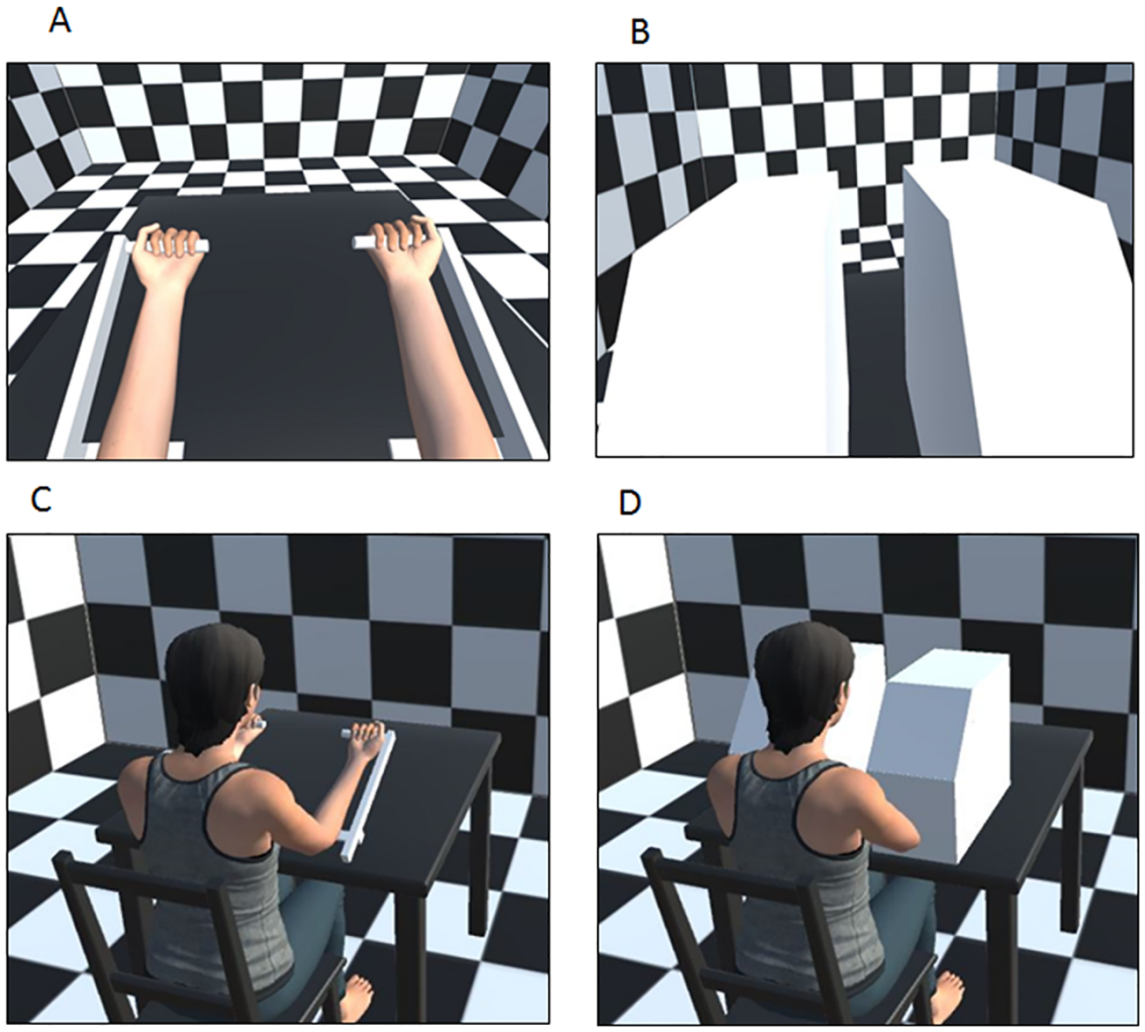
The virtual reality setup. The upper panels show the participant’s view (i.e. a first-person perspective) when the avatar’s forearms were visible (A) or masked (B). The lower panels show a third-person perspective of the virtual environment when the avatar’s forearms were visible (C) or masked (D).

In the “bimodal” condition, displacement of the participant’s left forearm was coupled in real time to displacement of the avatar’s forearms, both of which moved with the same angular amplitude and speed as the participant’s real left forearm (from either 15° to 45° or from 45° to 15°, relative to the horizontal plane). Hence, visual stimulation (displacement of the avatar’s forearms) was combined with real displacement of the left arm, as it would be in the “physical” mirror paradigm. In the “proprioceptive**”** unimodal condition, the avatar’s arms were masked by two virtual boxes on the virtual table (Fig 3). The 3D virtual room was still visible. Lastly, in the “visual” unimodal condition, the two avatar’s arms were flexed and extended at 3.8°/s for 8 s while the participant’s right and left forearms remained static and positioned at 30° (relative to the horizontal plane).

Whatever the experimental condition, participants were required to look at and pay attention to their virtual right forearm. The investigator checked that this was the case on the monitor showing the participant’s view of the virtual scene. Each experimental condition (the bimodal condition and each of the two unimodal conditions) was repeated eight times in blocks of four trials and in a pseudo-random order, giving a total of 24 flexion or extension trials. Each trial consisted in a baseline period of 5 to 10 seconds with static real and virtual arms followed by a 8 seconds period of real or virtual arm displacement. Following each trial, the participant was required to verbally rate the direction and speed of the illusory displacement experienced in its right static arm on an integer scale from 0 to 10. A rating of 0 corresponded to the absence of illusory displacement, and 10 corresponded to the same speed of displacement as for the passively moved left forearm or its avatar. The participant was also required to estimate the time (in seconds) to onset of the illusory displacement and the time to offset, that is, from the beginning of the arm displacement, ranging therefore from 0 seconds (no illusion) to 8 seconds (sustained illusion throughout the whole arm displacement). Once these different parameters had been rated, the next trial began. In order to become familiarized with these verbal ratings, the participant performed familiarization trials. The participant was allowed to rest for approximately five minutes after half of the 24 recorded trials had been performed.

### Data analysis

Three different measures were used to estimate and quantify the illusion.

Occurrence of the illusion: The occurrence of an illusion was noted whenever the participant reported an illusory displacement.
The Speed of illusory movement: This measure corresponded to the estimated speed of the illusion (from 0 to 10) recorded after each trial.
The duration of the illusion: The duration of the illusion corresponded to the time between the onset and offset of the illusory displacement expressed in percentage of time relative to the duration of the arm displacement.

### Statistics

The speed and duration of illusory movement were analysed in two-way repeated-measures analysis of variance (ANOVA) with three sensory conditions (*bimodal*, *proprioceptive*, *visual*) and two movement direction conditions (*flexion*, *extension*). The values stated by the participants were Huynh-Feldt corrected, and post-hoc tests were performed using Holm’s correction for multiple comparisons. One sample t-tests against zero were performed to test whether the illusion was present in the different sensory modalities. Pearson correlation analysis was performed to investigate the putative linear relationship between the observed illusions under the different experimental conditions. The threshold for statistical significance was set to p < 0.05.

## Results

A combination of vision of the displacement avatar’s forearms (flexion or extension) and passive displacement of the participant’s left forearm evoked a “virtual” mirror illusion of right forearm displacement in the same direction in 28 (82%) of the 34 study participants. Three participants experienced a kinaesthetic illusion in the opposite direction in some trials under the bimodal condition, whereas the remaining three participants failed to experience a kinaesthetic illusion in that bimodal condition. Given that the objective of the present study was to evaluate the respective contributions of visual and proprioceptive signals to the kinaesthetic mirror illusion, these six participants were excluded from further analysis. Hence, the final analysis included 28 participants.

### Occurrence of the kinaesthetic illusion

As mentioned above, the 28 participants included in the final analysis experienced—at least once over 8 trials—a kinaesthetic illusion in the bimodal condition, that is, when vision of the avatar’s moving forearms was combined with passive displacement of the participant’s left forearm. When each trial per sensory condition (n = 8) and each participant (n = 28) were considered (224 trials in total per sensory condition), the illusion occurrence rate reached 85% under the bimodal condition, 42.5% under the visual unimodal condition (i.e. when vision of the moving avatar’s forearms was not accompanied by passive displacement of the left forearm) and 24.5% under the proprioceptive unimodal condition (i.e. when vision of the avatar’s forearms was prevented during passive displacement of the participant’s left forearm) (Table 1).

**Table 1:** The mean ± SD occurrence, speed and duration of the kinaesthetic illusion under each of the experimental conditions.

	Bimodal		Unimodal			
	Vision + proprioception		Vision		Proprioception	
	Extension	Flexion	Extension	Flexion	Extension	Flexion
***Occurrence (%)***	85	85	47	38	27	22
***Speed***	6.3 ± 2.7	6.4 ± 2.9	2.9 ± 2.5	2.3 ± 2.3	1.1 ± 1.9	0.9 ± 1.9
***Duration (%)***	69 ± 30	70 ± 32	35 ± 34	28 ± 31	22 ± 32	19 ± 29

### The Speed of illusory movement (subjective rating)

**Fig 4:**
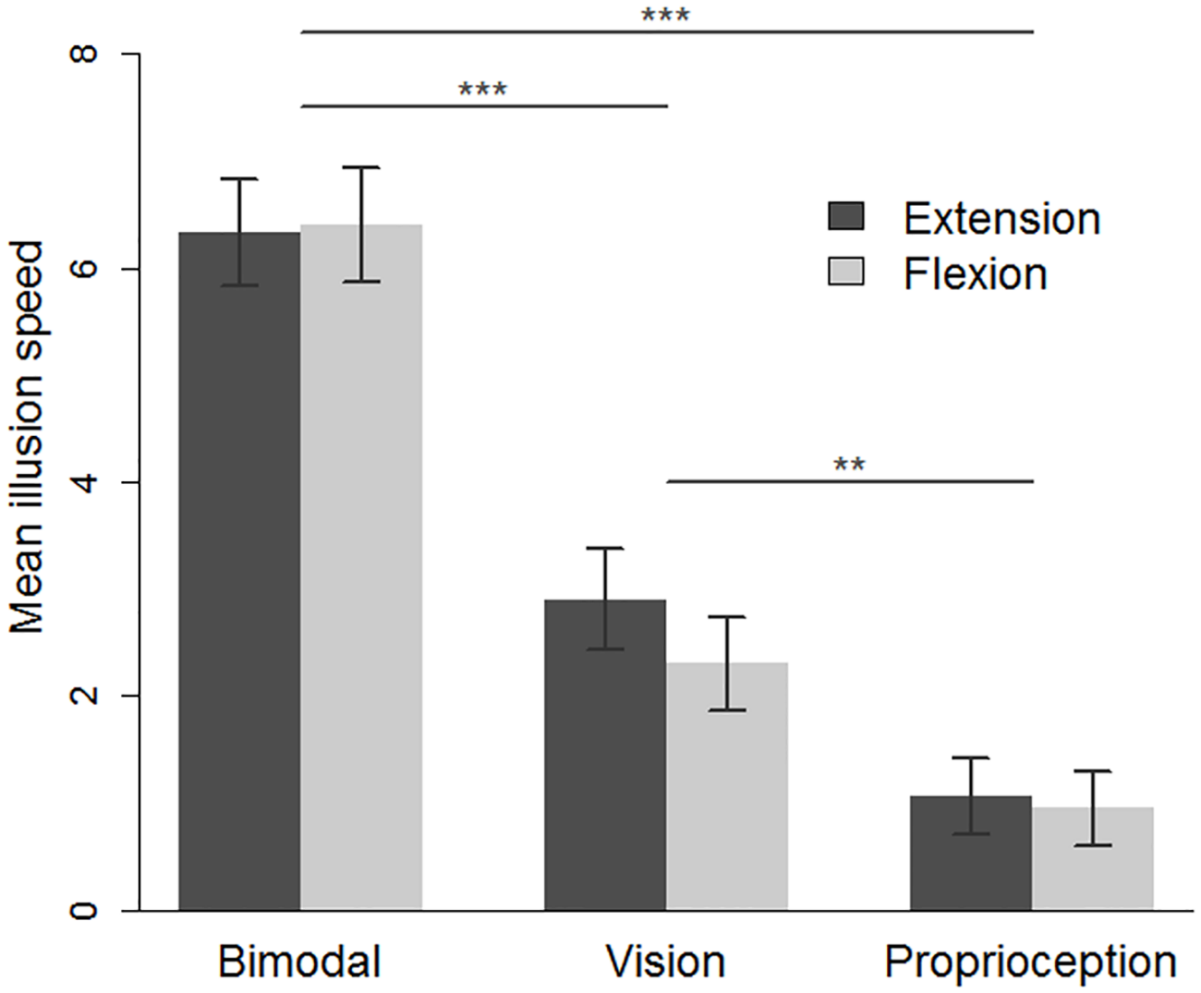
The mean speed of the illusion, as a function of the sensory condition (bimodal, vision, and proprioception) and the movement direction condition (extension and flexion). * p <0.05, *** p<0.001. The error bars correspond to the standard error of the mean.

As mentioned above, a rating of 0 corresponded to the absence of illusory displacement, and 10 corresponded to the same speed of displacement as for the passively moved left forearm or its avatar. As can be seen from Fig 4, the mean ± SD speed of the kinaesthetic illusion was higher in the bimodal condition (6.3 ± 2.7) than in the two unimodal conditions (vision: 2.6 ± 2.3; proprioception: 1 ± 1.7). Indeed, the ANOVA revealed a significant effect of the sensory condition (F_(2,54)_ = 53.2, p<0.0001, η^2^p = 0.66). A Holm post-hoc analysis confirmed that when the flexion and extension movements were pooled, the mean speed of the illusion was higher in the bimodal condition as compared to the unimodal visual condition (p<0.0001), the latter being higher than in the unimodal proprioception condition (p = .004). One sample t-test analysis showed that the mean illusion was significantly different from zero in the three sensory conditions (bimodal: t_(27)_ = 12.5, p<.001; vision: t_(27)_ = 6.001, p<.001; proprioception t_(27)_ = 3.2, p = .004) attesting that kinaesthetic illusion, though variable in terms of occurrence and speed, were present in the three sensory conditions. However, it must be mentioned that the sum of the illusion speeds observed in the two unimodal conditions was significantly smaller than that observed in the bimodal condition (t(27) = 4.5 p<.0001).

The mean ± SD speed of the illusion was similar for the extension (3.4 ± 1.72) and flexion (3.2 ± 1.70) movements as revealed by the absence of a significant main effect (F_(1.27)_ = 0.77, p = 0.38, η^2^p = 0.03). The interaction between sensory and direction of movement factors was not significant (F_(2,54)_ = 1.7, p = 0.18, η^2^p = 0.06).

Correlation analysis showed that the speed of the illusion in the visual unimodal condition appeared to be a much better predictor of the speed in the bimodal condition (r = 0.45, p = 0.015) than that in the proprioceptive unimodal condition was (r = 0.03, p = 0.87). The two unimodal conditions were not significantly correlated with one another (r = 0.12, p = 0.53).

### The duration of the illusion (expressed in percentage of time)

**Fig 5:**
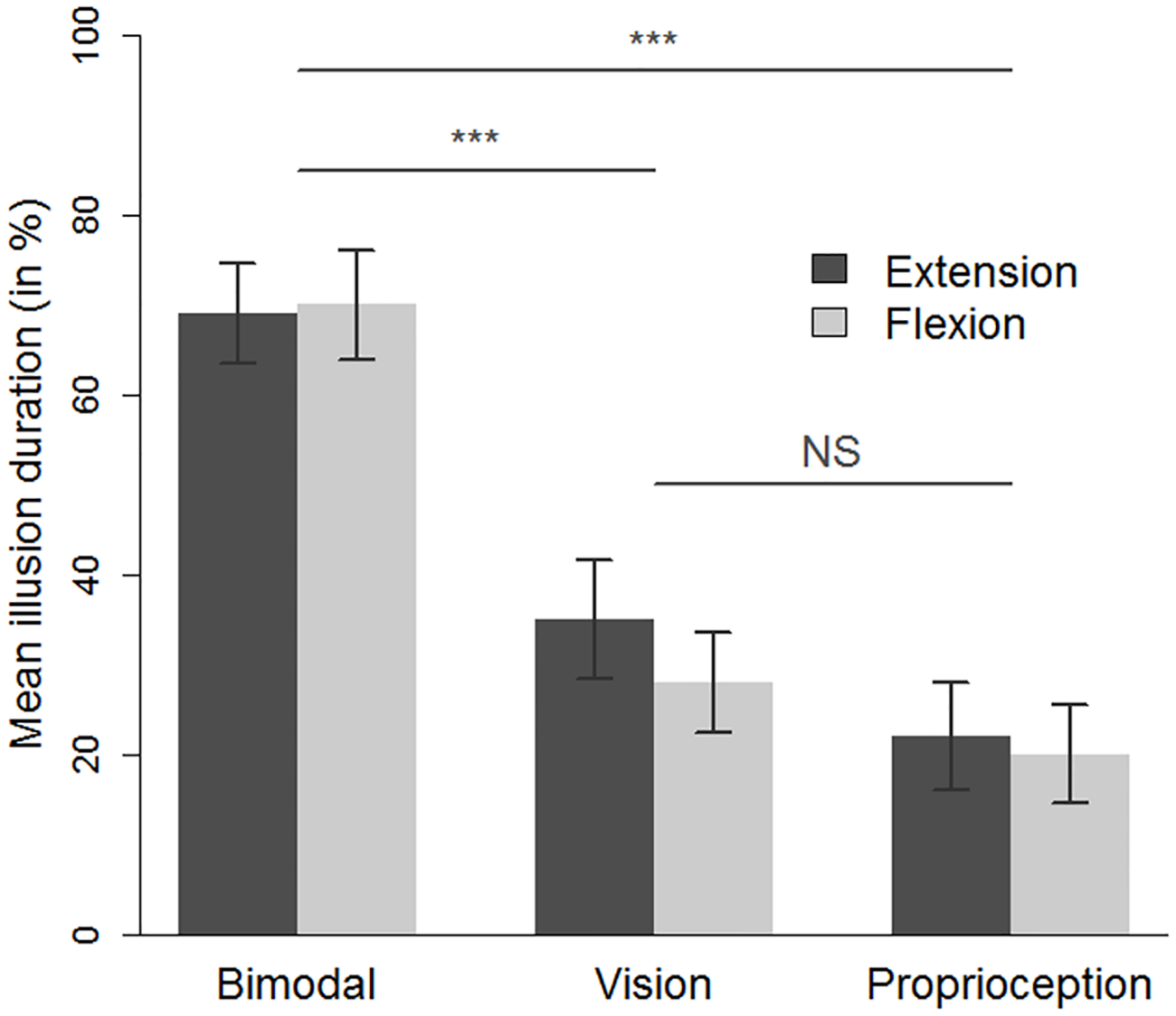
The mean duration of the illusion, expressed in percentage, as a function of the sensory condition (bimodal, vision, and proprioception) and the movement direction condition (extension and flexion). *** p<0.001, NS: non significant. The error bars correspond to the standard error of the mean.

Statistical analyses of the kinaesthetic illusion’s duration gave rather similar results to that related to the speed of the illusion (see Fig 5). Indeed, the ANOVA revealed a significant effect of the sensory condition (F_(2,54)_ = 29, p<0.0001, η2p = 0.52) with a mean ± SD duration of the kinaesthetic illusion longer in the bimodal condition (69.5% ± 30.4) than in the two unimodal conditions (vision: 31.5% ± 32; proprioception: 20.5% ± 28). A Holm post-hoc analysis confirmed that when the flexion and extension movements were pooled, the mean duration of the illusion was longer in the bimodal condition as compared to the two unimodal conditions (bimodal vs visual: p<0.0001; bimodal vs proprioceptive: p<0.0001). The mean duration of the illusion was not different between the two unimodal conditions (p = 0.12). Neither the main effect of movement (F_(1.27)_ = 1.1, p = 0.31, η2p = 0.04) nor the interaction between movement and sensory condition (F_(2,54)_ = 2.4, p = 0.09, η2p = 0.08) reached significance. As for the speed parameter, the sum of the illusion durations observed in the two unimodal conditions was significantly smaller than that observed in the bimodal condition (t_(27)_ = 2.1 p = .04).

As for speed analysis, correlation analysis showed that the duration of the illusion in the visual unimodal condition appeared to be a better predictor of the duration in the bimodal condition (r = 0.44, p = 0.02) than that in the proprioceptive unimodal condition was (r = 0.30, p = 0.11). The two unimodal conditions were not significantly correlated with one another (r = 0.21, p = 0.27).

## Discussion

### The real and virtual mirror paradigms

In the physical mirror paradigm, the participant sees the reflection of one arm in the mirror. This creates (by symmetry) a virtual opposite arm, and the real opposite arm remains out of sight. This visual arrangement creates vivid body illusions [19,20]; when the visible arm is moved (either actively or passively), its reflection in the mirror produces a kinaesthetic illusion of symmetrical bimanual movement (i.e. an illusory displacement of the static arm hidden behind the mirror) [10,21,22]. Here, both the image of the moved arm and its reflection in the mirror were replaced by the virtual arms of an avatar, as seen from a first person perspective. Our present results revealed that in most participants, the combination of passive displacement of the participant’s left forearm with visual displacement of the forearm’s avatars created a kinaesthetic illusion in their static (right) arm in the same direction of movement. This kinaesthetic illusion mimics the one classically reported in the “physical” mirror paradigm, and we shall refer to it henceforth as the “virtual mirror illusion”.

The proportion of our participants that failed to experience a virtual mirror illusion (n = 3, ~ 9%) was slightly smaller than the proportion (15–20%) that typically fails to experience a physical mirror illusion [13–15, 23]. Three other participants reported a reverse illusion on a few occasions (in 7 of the 264 trials), i.e. illusory displacements in the direction opposite to that visualized for the avatar’s forearms. The low proportion of these reverse illusions prevents us from drawing any meaningful conclusions.

### The virtual mirror illusion is not purely visual in origin

A large body of literature data attests to the strong involvement of visual signals in kinaesthesia in general and the mirror illusion in particular [8–9, 24–26]. The results of the present experiment do not challenge this view but show that the visual signals’ involvement in the mirror illusion—often considered as a prototypic visual illusion—is overstated. In the physical mirror paradigm, the reflection of an arm is inevitably accompanied by proprioceptive signals from the moved arm. These concomitance prevents any attempt to distinguish between the respective contributions of visual and proprioceptive signals to the genesis of illusion. Here, we used virtual reality to circumvent this methodological issue; the avatar’s forearms could be moved with or without concomitant movement of the participant’s left arm. Our results revealed that the kinaesthetic illusion was stronger (in terms of speed and duration) and more frequent when the displacements of the avatar’s forearms were combined with real passive displacement of the left arm than when the latter was static. Furthermore, kinaesthetic illusions (albeit weaker and less frequent) were reported when the avatar’s forearms were masked. Taken as a whole, our results are in line with the hypothesis whereby the kinaesthetic mirror illusion is not purely visual in origin but involves non-visual signals (namely bilateral proprioceptive signals).

The bimanual integration of proprioceptive afferents in both movement perception and motor control has been reported in the literature. Indeed, stimulating the muscle proprioceptive afferents of one arm affects both the motor behaviour of the other arm [27–30] and its perceived position and movement [16,17,31,32,33]. For instance, the proprioceptive processing error observed when participants are asked to point to their unseen hand [30,31,34] is lower when the two hands move together [30,31] than when only one hand moves. In the field of perception, Izumizaki et al. [16] and Hakuta et al. [17] showed that stimulating the muscle proprioceptive afferents of one arm (using tendon vibration) modified the perceived position of the other arm. Furthermore, we recently showed that once the mirror illusion has been evoked, it can survive visual occlusion for a few seconds (as long as the contralateral arm is still moving) and decays slowly. Taken as a whole, our past and present results confirm that kinaesthesia involves all the available sensory signals—including those originating from contralateral body segments.

### Should a real mirror box or a virtual mirror box be used in rehabilitation?

The mirror arrangement has been extensively used not only in rehabilitation wards but also in homecare programs for hemiplegic patients or for amputees suffering from phantom limb pain (for a critical review, see [11]). The mirror box’s low cost, low technical complexity, ease of use, and ability to resurrect phantom limbs all contribute to the popularity of this approach to rehabilitation [11]. Although a number of published studies have emphasized the effectiveness of mirror-like virtual displays for phantom limb pain [35] or motor recovery [36,37], virtual reality remains a highly technical, expensive tool that may not provide more clinical benefit than conventional rehabilitation protocols can ([38]; for a review, see [37]). With a view to rehabilitation, some facets of the virtual mirror box should nevertheless be considered. Firstly, clinicians know well that rehabilitation therapy may be lengthy when the patient looses interest. Virtual reality designs (including mirror-like setups) should be considered as adjuncts to standard care, and (with a view to improving the patient’s compliance) can be made far more entertaining than a physical mirror box increasing consequently patient’s compliance [38]. Secondly, patients must have one valid body segment for movement or for reflection in an actual mirror to operate. As a consequence, bilateral amputees or patients paralysed on both sides of the body cannot benefit from physical mirror therapy but might benefit from virtual mirror therapy; the participant would see two of the avatar’s arms or legs moving in the virtual scene. Considering the limited impact of visual stimulation not accompanied by concomitant proprioceptive (or motor) signals, a virtual reality display should nevertheless be combined with other signals (see [39]) (those originating from valid body segments, for example). Lastly, one possible advantage of virtual mirror therapy is that the avatar’s arms (or legs) are unlikely to be confused with real limbs. This might reduce the side effects of physical mirror therapy in (for example) patients who cannot bear to see a reflexion of a lost limb that they are still mourning

### Conclusion

Here, we reproduced the kinaesthetic mirror illusion in a virtual reality experiment. A combination of passive displacement of the left forearm with displacements of the avatar’s forearms induced a vivid illusory displacement of the static right arm in most participants. Our results also showed that the virtual image of a moving arm is not sufficient *per se* to systematically elicit strong “virtual” mirror illusions but is magnified by concomitant bilateral proprioceptive signals. Virtual reality could conceivably be introduced into rehabilitation wards as an adjunct to conventional treatments for motor rehabilitation, pain relief, etc. The potential benefits of virtual mirror therapy might be related to the intensity and quality of concomitant signals from the affected and/or healthy limbs, just as in the physical mirror illusion.
